# A quantum dot single-photon source with on-the-fly all-optical polarization control and timed emission

**DOI:** 10.1038/ncomms9473

**Published:** 2015-10-05

**Authors:** Dirk Heinze, Dominik Breddermann, Artur Zrenner, Stefan Schumacher

**Affiliations:** 1 Department of Physics, Center for Optoelectronics and Photonics Paderborn (CeOPP), Universität Paderborn, Warburger Strasse 100, 33098 Paderborn, Germany; 2 College of Optical Sciences, University of Arizona, Tucson, Arizona 85721, USA

## Abstract

Sources of single photons are key elements for applications in quantum information science. Among the different sources available, semiconductor quantum dots excel with their integrability in semiconductor on-chip solutions and the potential that photon emission can be triggered on demand. Usually, the photon is emitted from a single-exciton ground state. Polarization of the photon and time of emission are either probabilistic or pre-determined by electronic properties of the system. Here, we study the direct two-photon emission from the biexciton. The two-photon emission is enabled by a laser pulse driving the system into a virtual state inside the band gap. From this intermediate state, the single photon of interest is then spontaneously emitted. We show that emission through this higher-order transition provides a versatile approach to generate a single photon. Through the driving laser pulse, polarization state, frequency and emission time of the photon can be controlled on-the-fly.

## Abstract (web summary)

Single photon sources are important for applications in quantum information. Here, the authors exploit higher-order transitions from a biexciton state to the ground state of a semiconductor quantum dot to emit single photons with all-optical control of their frequency, polarization and emission time.

## Graphical abstract

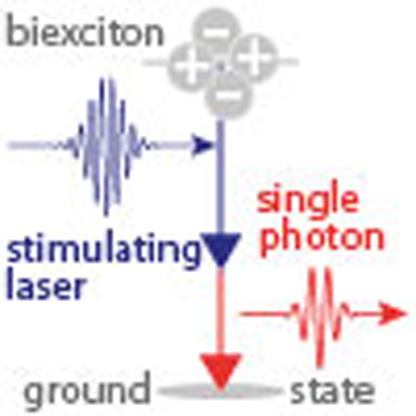

Semiconductor quantum dots have proven their promise as basic building blocks in various applications in the field of semiconductor-based quantum optics and quantum communication^1^. These semiconductor nanostructures have been used as well-controlled on-demand quantum emitters for single photons^2^,^3^,^4^,^5^,^6^,^7^,^8^,^9^ as well as for lasing at the single-photon level^10^,^11^ and to generate polarization entangled pairs of photons^12^,^13^,^14^,^15^,^16^,^17^,^18^. It has been demonstrated that the interaction of the quantum dot's electronic excitations with optical fields and the emission characteristics of photons can be tailored to a large extend by use of optical cavities^5^. Even on-chip solutions of quantum-dot cavity systems with build-in electrically pumped microlaser sources have recently been demonstrated^19^,^20^.

If a semiconductor quantum dot is excited from its electronic ground state, the lowest excited configurations are the exciton states with one electron–hole pair in the system. Through further excitation from either of the excitons, the biexciton state with two electron–hole pairs can be excited. These excited states are relatively long-lived with radiative lifetimes typically on the order of a nanosecond such that optical transitions can be studied in detail and also photon emission can be utilized efficiently. Most previous studies have focused on the emission of one or two subsequent (cascaded) photons from the biexciton to exciton or exciton to ground-state transition, respectively^18^,^21^. In contrast, recently it was noted that semiconductor quantum dots can also efficiently couple to an optical light field through a direct two-photon transition from ground state to biexciton and vice versa^22^. Both of these states are spin-zero states such that a direct two-photon transition is allowed and efficient. Fully stimulated coherent two-photon excitation has been demonstrated in both degenerate^23^ and two-colour^24^ scenarios. On the other extreme, a fully spontaneous two-photon emission was reported^25^ and explored^26^,^27^.

Alternatively, a mixed scenario can be analysed for the two-photon emission from the biexciton: one photon is stimulated, the other one spontaneously emitted. In analogy to a partially stimulated down-conversion process^28^, the first photon is triggered/stimulated by an external pump laser field. This field can be off-resonant to all one-photon active transitions such that it drives the system between the biexciton state and a virtual level inside the system's band gap. However, then the first photon only gets actually emitted if a second photon is spontaneously emitted (possibly into a cavity mode) and bridges the remaining energy gap to the ground state. Given the first photon's stimulated nature, its properties are determined by the stimulating pump. Following from fundamental energy and spin conservation, the second photon has complementary properties such as polarization and frequency. Therefore, changes in the parameters of the pump laser would allow for all-optical control and on-the-fly changes to the properties of the emitted single photon.

Here, we present a detailed theoretical analysis for a quantum-dot cavity system and show numerically that a single-photon source as discussed above can be realized for a wide range of realistic system parameters. Our calculations show that the properties of the emitted single photon (as a true quantum object) can indeed be all-optically controlled with the classical laser field triggering the emission. We show that control of polarization state, frequency and time of emission of the single photon can be achieved on a picosecond timescale with the emission event inside the short-time window marked by the presence of the triggering pulse. This gives us all-optical control over the single-photon emission.

## Results

### Setup and emission scheme

**Figure 1:**
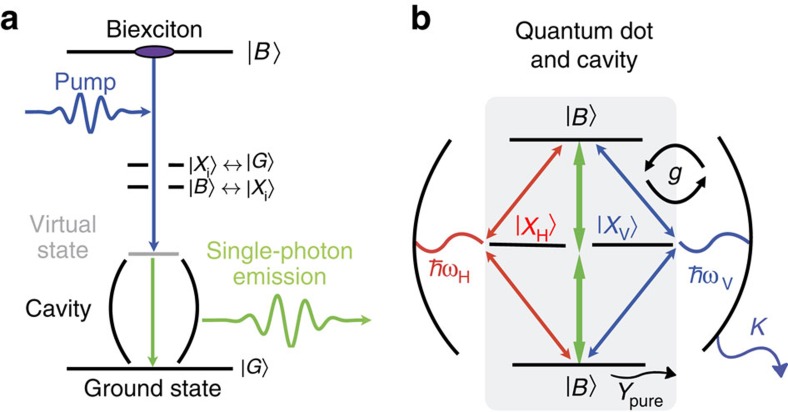
Single-photon generation through two-photon emission from a quantum-dot biexciton. (**a**) Initially the quantum dot is in the biexciton state |*B*〉. Emission of the first photon is triggered by the pump laser field in a stimulated emission process into a virtual state inside the bandgap. The second photon is emitted spontaneously and is channeled into a cavity mode when the quantum dot relaxes to its ground state |*G*〉. The second photon, which is the single photon of interest here, has properties such as polarization and frequency complementary to the stimulating pump laser field. Both stimulating laser and cavity are off-resonant to the quantum dot single-photon transitions involving the exciton states |*X*_*i*_〉 with horizontal, *i*=H, and vertical, *i*=V, polarization. (**b**) Illustration of the quantum-dot cavity system analysed. The electronic system of the quantum dot is coupled to the photonic modes of the cavity with coupling strength *g*. The cavity mode frequencies are *ω*_H_ and *ω*_V_. Photon loss from the cavity occurs with rate *κ* and electronic coherence is lost on the timescale 1/*γ*_pure_.

The microscopic many-particle theory we use in our analysis is based on the quantum-dot cavity model illustrated in Fig. 1. Included are the relevant electronic configurations of the quantum dot. These are ground state |*G*〉, excitons |*X*_H_〉 and |*X*_V_〉 and biexciton |*B*〉. The electronic system is coupled to the photons in two cavity modes with frequencies *ω*_H,V_ with coupling strength *g*. The two orthogonal linear polarization directions are denoted as horizontal (H) and vertical (V). In addition to the quantized light fields in the cavity modes, an off-resonant coherent laser field is included to trigger the photon emission. For a given initial state and given external laser field, we evaluate the dynamical evolution of all populations and coherences in the system. In particular, we extract detailed information about the photon emission from the system. Photon loss from the cavity with a rate *κ* and loss of electronic coherences on the relevant timescale ∼1/*γ*_pure_ are included. Full details on the theoretical approach are given in the Methods section below.

### Single-photon generation from a two-photon process

**Figure 2:**
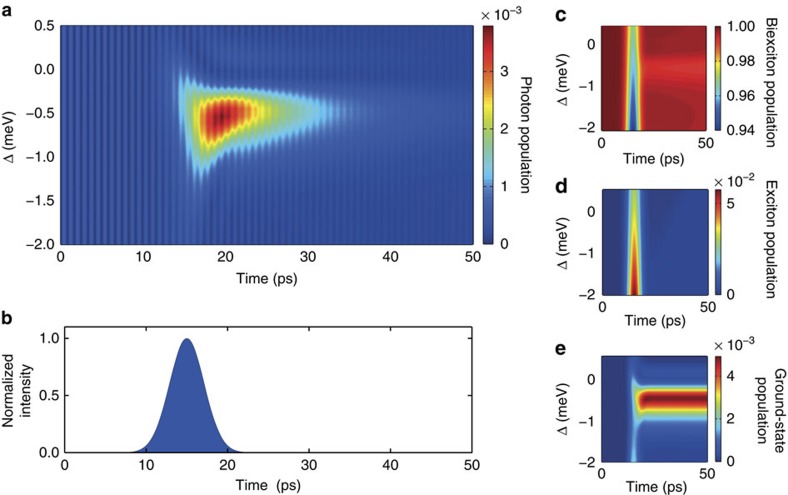
All-optical control of the single-photon emission event. (**a**) Computed time-resolved photon population inside the cavity for different detunings Δ of the laser pulse from the two-photon resonance condition. (**b**) Temporal intensity profile of the stimulating laser pulse triggering the single-photon emission. Photon emission into the cavity mode in **a** occurs during the presence of the stimulating pulse. The optimum detuning from the two-photon resonance condition is radiatively shifted to Δ≈−0.54 meV as discussed in detail in the text. (**c**–**e**) Computed populations of the electronic states. Shown are the biexciton (**c**), exciton (**d**) and ground-state (**e**) population, respectively.

To study the scheme outlined above and illustrated in Fig. 1a, initially we prepare the quantum dot in the biexciton state with no photons in the cavity. Recent studies have shown the robust initialization of the biexciton^18^,^29^. Then a picosecond light pulse is applied, driving the system between the biexciton and a virtual state inside the band gap. When the pulse frequency *ω*_L_ is tuned such that the two-photon resonance condition from ground to biexciton state is fulfilled, *E*_B_−*E*_G_=*ℏ*(*ω*_L_+*ω*_H,V_), energy conservation allows spontaneous emission of a single photon into the cavity mode. To increase the probability of the emission event to occur during the presence of the stimulating pulse, first we use a high-quality cavity with *κ*=*ℏ*/10 ps^−1^ (quality factor *Q*≈21,000 at 880 nm wavelength) and a coupling strength of the single-photon transitions to the cavity mode of *g*=*ℏ*/10 ps^−1^≈66 μeV. For these parameters, Fig. 2a shows the computed time-resolved photon population (defined as 
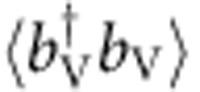
 with the cavity photon creation and annihilation operators, 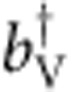 and *b*_V_, respectively) in the V-polarized cavity mode for different detunings of the V-polarized pulse from the two-photon resonance condition. The envelope of the 5-ps pulse with peak Rabi energy of *Ω*_0_=1.5 meV is depicted in Fig. 2b. The result in Fig. 2a clearly shows that a photon is emitted when—and only when—the (here non-degenerate) two-photon resonance condition for laser pulse and cavity mode at detuning Δ=*E*_B_−*E*_G_−*ℏ*(*ω*_L_+*ω*_V_)≈0 is met. We note that we observe a slight field-induced shift of the emission from the bare two-photon resonance to Δ≈−0.54 meV. The resonance condition is radiatively shifted because of the presence of the strong laser pulse triggering the emission. In the limiting case of a long pulse stimulating the emission process and a system that is fully inverted to the biexciton state, we analytically find a modified resonance condition with optimum pulse detuning 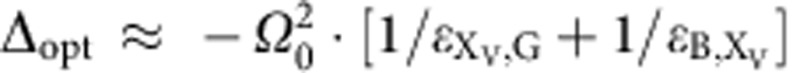 with *ɛ*_*α*,*β*_=*E*_*α*_−*E*_*β*_−*ℏω*_V_. Comparison with the optimum detuning numerically found also for pulses of finite length and different peak Rabi energies shows good agreement (see Supplementary Fig. 1 for details). In Fig. 2, we find that photon emission only occurs during the presence of the laser pulse. This is evidenced by the dynamical build-up of a finite photon population inside the cavity mode when the pulse is switched on. After the pulse has passed, the remaining photons get emitted from the cavity on the 10-ps timescale determined by the cavity loss *κ*. We note that in addition to the main emission feature, weak oscillations are visible as vertical stripes in Fig. 2a from the emission from the biexciton to exciton transition into the off-resonant cavity mode.

The dynamics of the biexciton, exciton and ground-state populations are depicted in Fig. 2c–e. Clearly visible is the adiabatic following of these populations during the presence of the off-resonant pump pulse. However, only for the frequency window close to the two-photon resonance condition, the ground-state and biexciton populations are changed by a sizeable amount after the stimulating pump pulse has passed. We stress that not a significant amount of population is generated in the exciton state(s) as the emission is strongly dominated by the direct two-photon channel.

We note that the absolute probability for the photon emission to occur and with it the potential brightness of the source can be further optimized by increasing pump intensity and pulse length along with other system parameters such as biexciton-binding energy, cavity frequency, coupling strength and cavity quality. In this context, it is important to note that the proposed scheme benefits from resonance enhancement of the (partly stimulated, partly spontaneous) two-photon emission when the pulse-induced virtual state, representing the stimulated part of the emission, is created spectrally close to the single-photon transitions. In the limiting case of long pulses, we analytically find that the photon emission from the desired process scales with 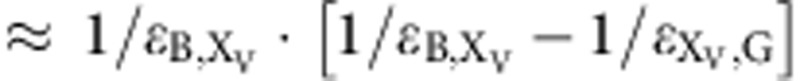. We further note that for higher pumping intensities and stronger couplings the system dynamics can also become more complex. True benchmarking of the potential performance will be left for a future study possibly along with an experimental demonstration. We note that for the system parameters chosen here, by increasing the pulse length by a factor of 10, to a pulse length of 50 ps, we find an almost linear increase of the emitted photon population with pulse length while still only generating an insignificant amount of exciton population.

### Optical polarization control of single-photon emission

**Figure 3:**
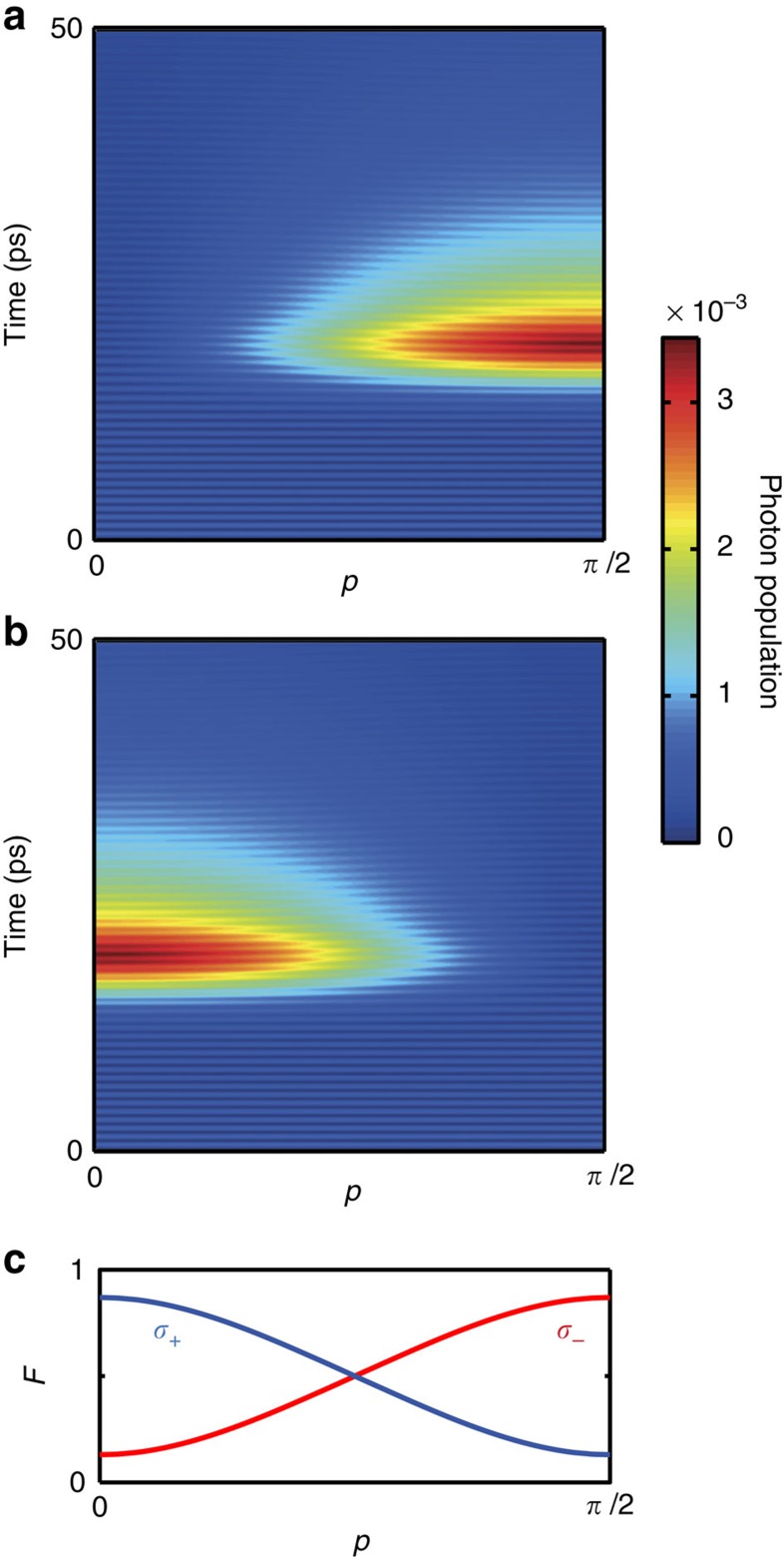
Optical polarization control of the single-photon emission. Shown is the time-resolved (**a**) minus (−) and (**b**) plus (+) circularly polarized photon population inside the cavity for different polarization states of the stimulating pump pulse triggering the emission. The pump polarization is parametrized as given in the main text such that variation of the parameter *p* from 0 to *π*/2 leads to a transition from − circularly (***σ***_−_, *p*=0) to + circularly (***σ***_+_, *p*=*π*/2) polarized pump. (**c**) Time-averaged normalized fraction *F* of − circularly and + circularly polarized photon populations. (**a**–**c**) Calculations are for the same parameters as in Fig. 2. Pump frequency is tuned to the optimum detuning with maximum emission at Δ=−0.54 meV.

In Fig. 3, we demonstrate that with the polarization state of the pump laser triggering the emission, the polarization state of the emitted photon can be controlled. The pump electric field amplitude and polarization state are parametrized as **E**=*E*_0_·(cos(*p*)***σ***_−_+sin(*p*)***σ***_+_), here with the real-valued parameter *p*. The limiting case *p*=0 (*p*=*π*/2) corresponds to light in the circular polarization state with polarization vector ***σ***_−_ (***σ***_+_). System parameters are the same as in Fig. 2. According to spin selection rules, when the pump is plus (+) circularly polarized, the emitted photon is minus (−) circularly polarized and vice versa. Figure 3a,b shows the time-resolved and Fig. 3c shows the time-averaged normalized (averaged from 12 to 45 ps and normalized at each point in time to the total cavity photon population) fraction *F* of photon population in the circularly polarized states. With changing the polarization parameter *p* of the stimulating pump from 0 to *π*/2, the expected (almost sinusoidal) change in the polarization state from + circularly to − circularly is found. The achievable contrast is slightly reduced by the spontaneous biexciton decay through the exciton states. These results give unambiguous evidence that the emitted photon stems from a spontaneous emission into the cavity mode and cannot result from photons pumped into the system through the laser source. By changing the polarization state of the pump anywhere in between the − and + circular polarization state (in general elliptically polarized), any polarization state can be realized for the emitted single photon of interest. Therefore, the classical laser field of the pump can be used to control the polarization state of a single photon as a true quantum object. In a scenario where also frequency filtering is applied during photon detection, the contrast for this polarization control could reach near 100% (no background photons are emitted in the spectral range of interest). To achieve efficient polarization control, the cavity modes must be degenerated (as can be realized in a micropillar cavity, for example), however, it is important to note that no frequency fine-tuning of cavity modes, for example, through temperature is needed as the stimulating laser can be tuned as needed.

### Optically timing the single-photon emission

**Figure 4:**
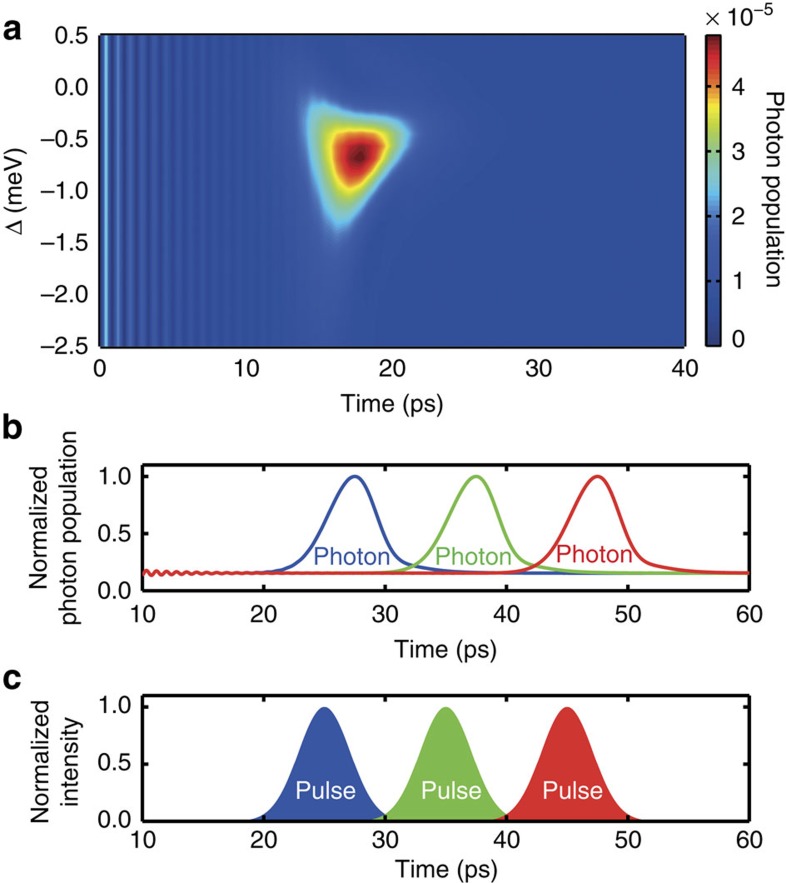
Controlling the time of emission with the arrival time of the pump. A lower-quality cavity with lower coupling strength with *g*/*κ*=0.04 is used. (**a**) The 5-ps pump pulse is centred at 15 ps. For a broad range of pump frequencies, a build-up of photon population and with it photon emission from the cavity is observed. In **b**, we demonstrate that the timing of the photon emission can be controlled by varying the arrival time of the pump. For a pump detuning of Δ=−0.66 meV, the normalized photon population in the cavity is shown for pump arrival at 25, 35 and 45 ps, respectively. (**c**) Normalized pump intensity corresponding to the different arrival times used in panel **b**.

In Fig. 4, we demonstrate that the single-photon emission can be timed with the arrival time of the stimulating pump. We also show that the scheme proposed here does not rely on the strong coupling or the high quality of the cavity mode used above. Here we present results obtained with a much lower coupling strength and in a low-quality cavity with *g*/*κ*=0.04 with *g*=1/50 ps^−1^≈13 μeV and *κ*=1/2 ps^−1^ (*Q*≈4,200). As shown in Fig. 4a, in this case, the cavity resonance condition is alleviated such that also the two-photon resonance condition is smeared out. This leads to a significant emission of the photon for a wider range of pulse detunings Δ. We also find that with the photons not being stored in the low-quality cavity for a long time, the photon population (which in this case roughly correlates with the actual emission dynamics) occurs inside the cavity only during the presence of the short 5 ps pulse with peak Rabi energy of 1.5 meV. This allows us to control the time of emission of the photon generated. This is explicitly shown in Fig. 4b,c for three different arrival times of the pump pulse. In Fig. 4b, a background in photon population is observed from the competing gradual decay of the biexciton through the biexciton to exciton transition. If desired, using spectral filtering these background photons can be eliminated in the detection^30^. The lower limit of the time resolution with which the emission time can be controlled is given by the lifetime of the photons inside the cavity and the minimum pulse length that can be used to efficiently trigger the emission process. For the parameters used here, the spectral separation of the different single- and two-photon resonances in the system limits the triggering pulse length to approximately 1 ps, for which the emission can still be selectively triggered (see Supplementary Figs 2 and 3 and Supplementary Discussion for details). We note that for larger detunings also shorter triggering timescales are possible. For excitation with degenerate two-photon Rabi-flopping from the ground state, selective optical biexciton initialization in typical InAs/GaAs quantum dots can also be achieved down to timescales of approximately 1 ps. An example of a combined initialization and emission sequence is shown in Supplementary Fig. 4. Previous approaches to trigger the emission of single photons from semiconductor quantum dots based on resonant excitation are limited to timescales typical for (Purcell enhanced) spontaneous decay^2^,^8^,^31^,^32^.

## Discussion

In recent experiments utilizing coherent resonant Rayleigh scattering in the low-intensity regime, single-photon emission with ultra-narrow emission lines given by the spectral properties of the laser source was reported^34^,^33^. Pulsed resonance fluorescence from a single-quantum dot exciton based on state preparation by Rabi-flopping was recently demonstrated to minimize linewidth and dephasing effects in the generation of indistinguishable photons^8^. Both schemes deliver single photons at the fixed exciton-to-ground-state transition frequency and polarization control of the emission would only be possible within the limits set by exciton fine-structure splitting.

In the present paper, we analyse the emission of a single photon into a cavity mode, which is introduced to enhance the probability of the photon to be emitted during pulsed stimulation. However, we would like to emphasize that, in general, the physical scheme introduced here does not rely on the presence of the cavity mode. In the presence of a cavity mode, frequency control of the emitted photon (which is inherent to the process studied) can only be efficiently performed in the spectral range given by the width of the cavity line. However, for emission into free space within the same scheme, the cavity resonance restriction would be completely eliminated. Then the emission frequency could be all-optically tuned with the frequency of the stimulation pump. The available spectral range would be limited by other exciton resonances (higher exciton shells and LO-phonon assisted processes) present in the system. For the specific two-photon emission process utilized here, the photon emission rate is second order in the coupling constant *g* and second order in the Rabi energy *Ω*_0_ of the light field triggering the emission. As a consequence, efficiency of the desired process would be reduced in free space such that control on ultrafast timescales may be difficult to achieve. However, we would like to re-iterate that other advantages of the proposed scheme such as all-optical frequency and polarization control for the photons would still be significant stengths. Using a cavity (with *g* smaller than both cavity and pulse width), the emission linewidth is given by the cavity or pulse width, whichever is smaller. In free space and for a sufficiently narrow laser line, the linewidth of the emission would be ultimately limited by the homogeneous linewidth of the electronic transitions (which may be radiatively limited) while at the same time offering full flexibility to optically control other important properties of the emitted photon. In particular, polarization control is not affected here by exciton fine structure splitting as the single exciton levels are not resonantly involved in the emission process. In the true strong coupling regime, *g*>*κ*, a more complex emission dynamics and spectral properties are observed that would be interesting to explore in the future.

In conclusion, we have proposed and analysed theoretically a new scheme for single-photon generation with semiconductor quantum dots. This scheme utilizes a partially stimulated two-photon emission from the quantum-dot biexciton. In this scheme, properties (polarization state, frequency, time of emission) of the spontaneously emitted single photon can be controlled on-the-fly and all-optically with a classical laser field. Future possibilities include using the same scheme for emission into free space such that frequency can be controlled in a wider spectral range. Applying a chirp in frequency and/or polarization state of the pump to control the complex temporal dynamics of the single-photon emission event could also be of great interest. An experimental realization of the proposed scheme would be highly desirable. We envision that the scheme we propose is promising for realization of the next generation of versatile quantum-dot-based single-photon sources.

## Methods

### Theoretical approach

We include the relevant electronic configurations of the quantum dot in our theory. These are ground state |*G*〉, excitons |*X*_H_〉 and |*X*_V_〉 and biexciton |*B*〉. The electronic system is then coupled to the photons in two cavity modes with orthogonal polarizations and frequencies *ω*_H,V_, in the two orthogonal polarization states denoted by H and V. In addition to the quantized light fields in the cavity modes an off-resonant coherent laser field is included to trigger the emission. In rotating wave approximation, the many-particle system Hamiltonian then reads:


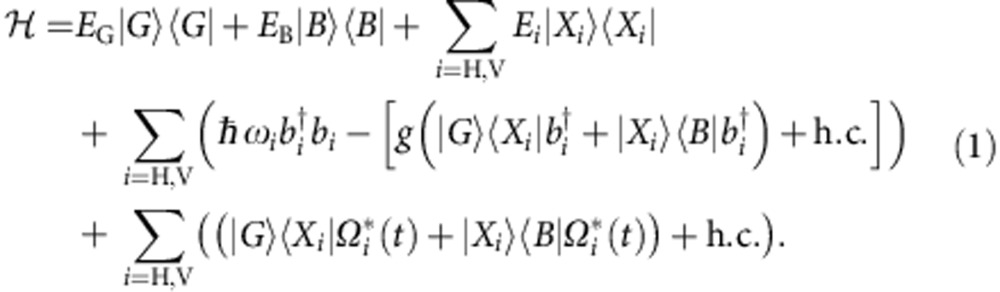


Here, 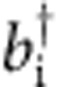 (*b*_*i*_) denote creation (annihilation) operators of photons in the cavity modes H and V and *Ω*_*i*_(*t*) gives the Rabi energy of the time-dependent coherent laser field projected onto the respective transitions with *i*-polarization. We note that no process is included in our hybrid theory such that the laser field could directly pump photons into the cavity modes. This approximation is well justified as in all our evaluations, the laser field is off-resonant to the cavity modes by several meV. The coupling strength of the electronic system to the cavity modes is given by *g*. The time-evolution of the system density operator *ρ*_*s*_ obeys the following equation of motion:


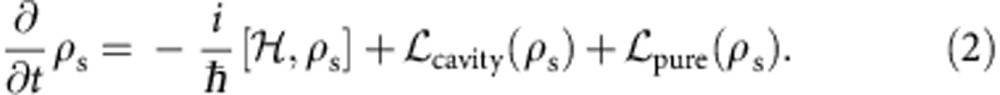


Coupling of the system to the environment is included through the two dissipative terms 
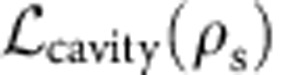
 and 
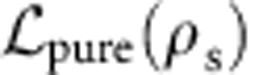
. The finite lifetime *ℏ*/*κ* of the photons inside the cavity is taken into account through the Lindblad term


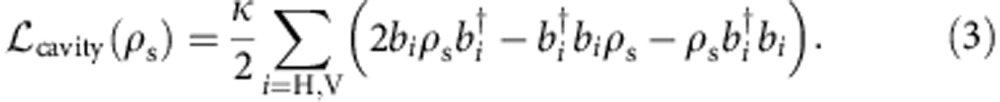


A pure dephasing of coherences between electronic configurations is included through


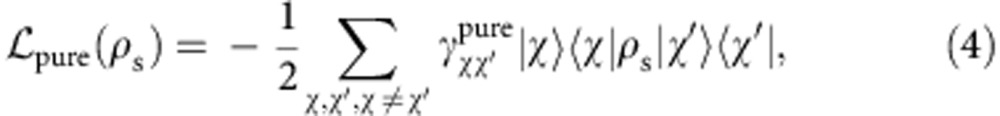


with *χ*,*χ*′∈{*G*,*X*_H_,*X*_V_,*B*} (ref. 35). We use the same value 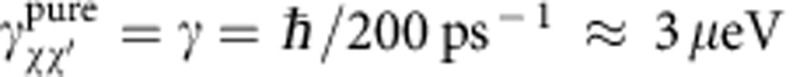 for all electronic coherences, which is a realistic value for pure dephasing of excitonic coherences at low temperature^36^,^37^. We note that long-lived coherences are not a prerequisite for the scheme introduced here, our results are qualitatively robust even with a much faster pure dephasing. A detailed analysis is given in Supplementary Fig. 5 and Supplementary Discussion. Fine structure splitting between exciton levels—typically of the order of several tens of μeV—does only cause minor quantitative changes to the results and with *E*_H_=*E*_V_ is set to zero for simplicity. Degeneracy is assumed for the cavity modes, *ω*_H_=*ω*_V_, which is needed for efficient polarization control. A reasonable biexciton-binding energy of 3 meV is assumed and the cavity modes are tuned 5 meV to the red of the biexciton to exciton transitions. Supplementary Fig. 2 shows a sketch of the relative spectral positions and line widths used. In this work, we assume the system to be initially in the biexciton configuration with no photons inside the cavity. For this initial condition, the system dynamics is obtained by explicitly solving equation (2) in the finite-dimensional Fock-space spanned by the degrees of freedom of our system. Numerical convergence of the results is typically achieved including states with up to two photons per cavity mode in the evaluations. Expectation values of any operator 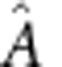 are computed by taking the trace with the system density operator, 
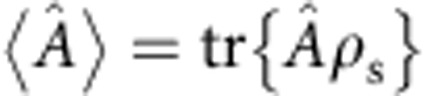
. We note that for simplicity, some secondary effects that could reduce the quantum efficiency of the single-photon emission have not been considered: phonon-assisted transitions into cavity mode or laser field are expected to be weak for the detuning of several meV used^38^. Radiative loss into other decay channels does not affect the rate of the single-photon emission process introduced here. However, we note that it plays a quantitative role and reduces the quantum yield if significant depopulation of the biexciton state occurs within the time until the emission is triggered. A quantitative analysis is given in Supplementary Fig. 6 and Supplementary Discussion.

## Additional information

**How to cite this article:** Heinze, D. *et al*. A quantum dot single-photon source with on-the-fly all-optical polarization control and timed emission. *Nat. Commun.* 6:8473 doi: 10.1038/ncomms9473 (2015).

## Supplementary Material

Supplementary Information Supplementary Figures 1-6, Supplementary Discussion and Supplementary References
