# Supplementary material for: A quantum dot single-photon source with on-the-fly all-optical polarization control and timed emission
Source: Nat Commun. 2015 Oct 5;6:8473. doi: 10.1038/ncomms9473 (PMC4600753; doi:10.1038/ncomms9473)
Supplement: Supplementary Information — Supplementary Figures 1-6, Supplementary Discussion and Supplementary References [file ncomms9473-s1.pdf]

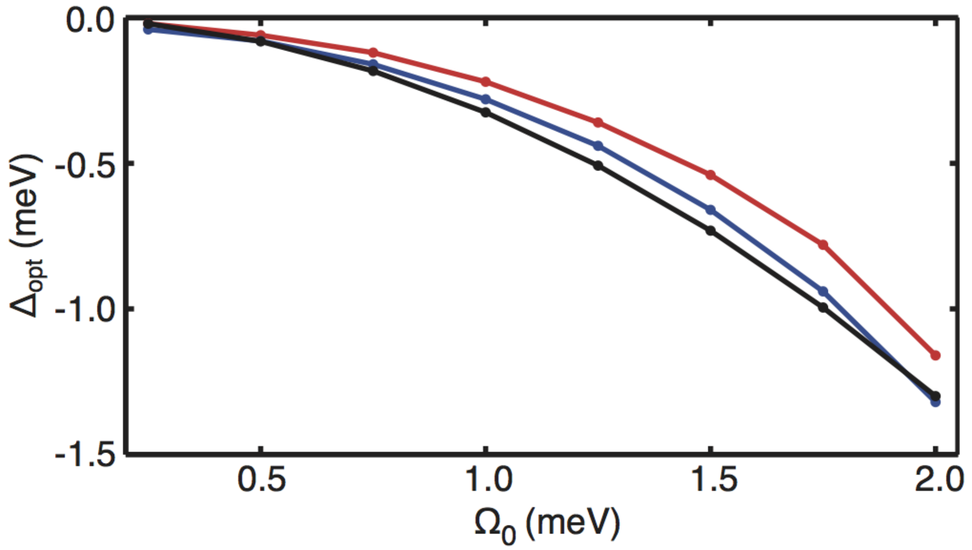

Supplementary Figure 1. Light-induced shift of resonance condition. Shown is the excitation-induced shift Δ_{opt} of the triggering pulse from the bare two-photon resonance condition Δ=0 for varying peak Rabi energy Ω_0 of the pulse. The red line corresponds to the parameters of Fig. 2 and the blue line to the parameters of Fig. 4. For comparison, the analytic result derived for the long-pulse limit is included (black line).

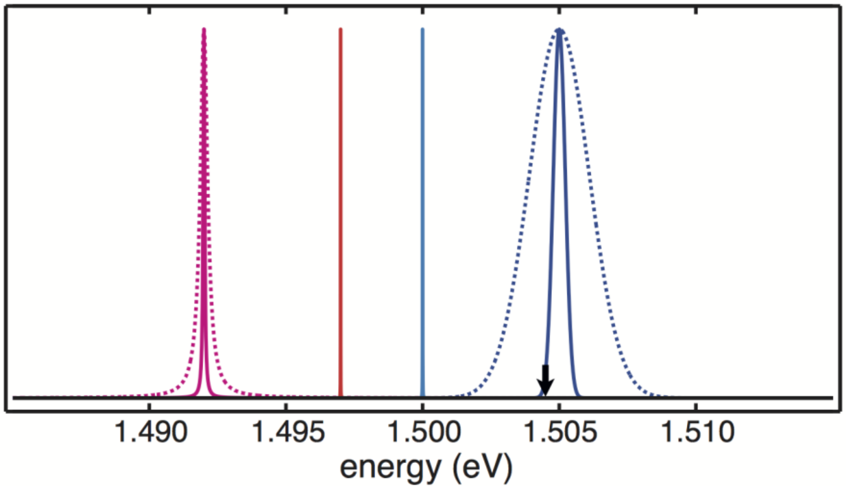

Supplementary Figure 2. Sketch of resonance and pulse energies and widths. The single-photon transitions are centered at 1.5 eV for the exciton to ground state (light blue solid line) and at 1.497 eV for the biexciton to exciton transition (red solid line), respectively, with a homogeneous linewidth of γ_{pure} = 3 μeV. For the cavity resonance at 1.492 eV two different linewidths of κ = 66 μeV as used for the results of Fig. 2 (magenta solid line) and 329 μeV as used for Fig. 4 (magenta dotted line) are shown. A Gaussian pulse with central frequency of 1.505 eV and FWHM intensity of 5 ps (dark blue solid line) and 1 ps (dark blue dotted line) is shown. The optimum pulse frequency as found in Fig. 2 including the light-induced shift is indicated by the black arrow.

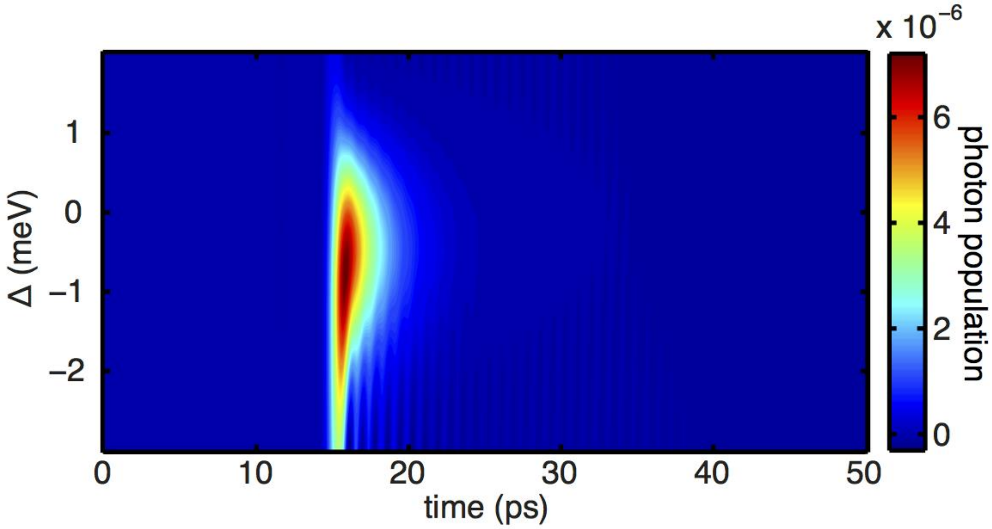

Supplementary Figure 3. Emission triggered with a short pulse. Parameters are as in Fig.4a with a 1-ps pump pulse used here. Shown is the difference in photon populations with and without pulse for varying detuning Δ from the bare two-photon resonance**.**

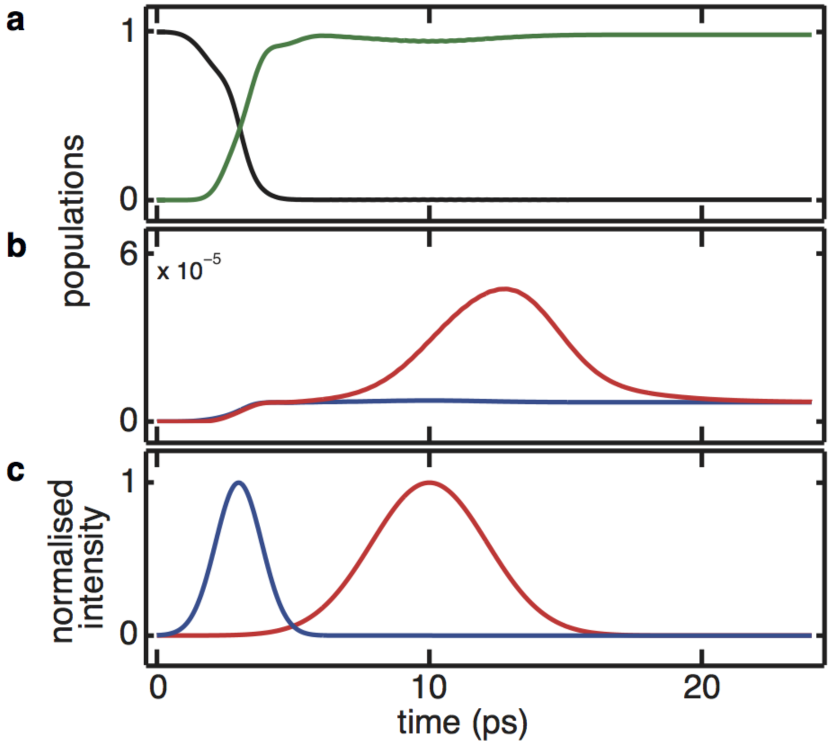

Supplementary Figure 4. Initialization and emission cycle. The initialisation is through a degenerate two-photon Rabi flop from the ground state to the biexciton state with a 2 ps pulse. The emission is triggered by a 5 ps pulse for the same system parameters as in Fig. 4. (**a)** population of the ground state (black line) and the biexciton state (green line). (**b)** photon population in cavity mode H (blue line) and cavity mode V (red line). (**c)** normalised intensities of the initialisation (blue line) and the stimulating pump pulse (red line).

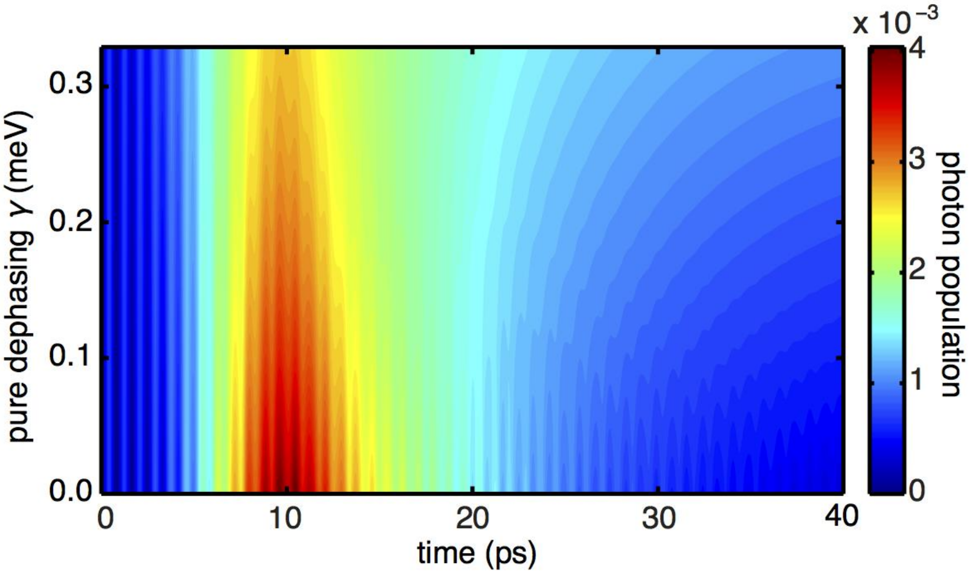

Supplementary Figure 5. Influence of pure dephasing on photon emission. Shown is the cavity photon population for varying pure dephasing γ for parameters as in Fig. 2 with optimum pulse detuning of Δ=-0.54 meV.

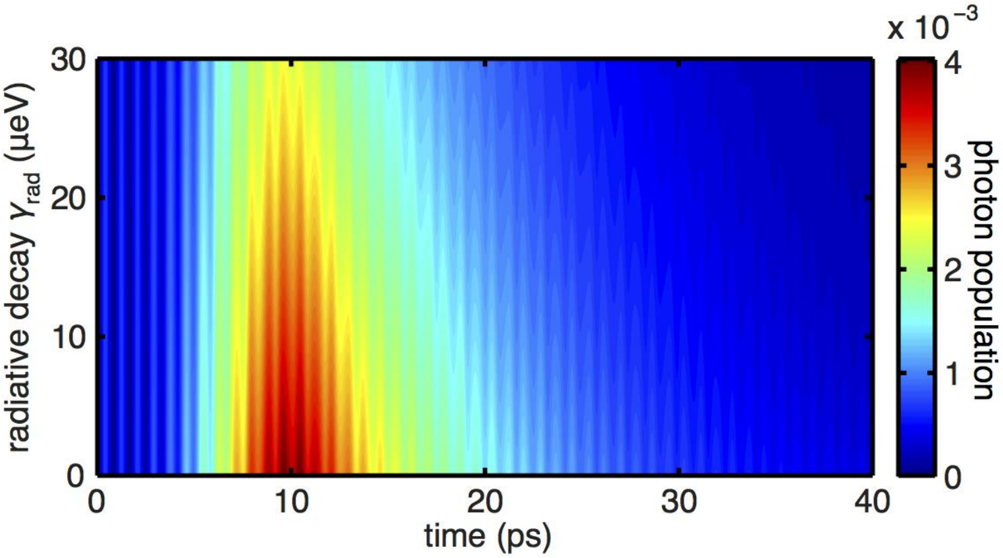

Supplementary Figure 6. Influence of radiative loss on photon emission. Shown is the cavity photon population for parameters as in Fig. 2 with optimum pulse detuning of Δ=-0.54 meV for varying radiative decay γ_{rad}.

Supplementary Discussion

1. Timing of the emission

A lower limit for the timing of the emission process achievable is set by the spectral width of the pulse in comparison with the separation of transitions illustrated in Supplementary Fig.2. If significant spectral overlap of the pulse is obtained with the single-photon resonances, the desired emission process can not be triggered efficiently in isolation. To explore this limit better, we have performed a calculation for the parameters of Fig.4 of the main text using a 1ps pulse to trigger the emission. The data is shown in Supplementary Fig.3. For the parameters used, if the pulse is much shorter than 1ps, especially for negative detuning $\Delta<0$ spectral overlap of the pulse with the biexciton to exciton transition is present such that the two-photon emission channel cannot be triggered in isolation anymore.

In Supplementary Fig.4 an optical initialization and emission cycle is shown. The biexciton initialization is done optically through a two-photon Rabi flop from the ground state. Photon emission is triggered shortly after, such that the full cycle is inside a time window of about 10ps.

2. Decoherence and radiative loss

Supplementary Fig.5 shows the cavity photon population for the system parameters of Fig.2 of the main text for optimum pulse frequency and varying pure dephasing. The pure dephasing is varied up to values that are realistic for elevated temperatures up to 150K [1,2]. The result demonstrates that pure dephasing at low temperatures is not limiting the emission scheme as it does not rely on long-lived coherences.

Below we adress the role that radiative loss from the biexciton into photonic modes not explicitly considered can play. This can best be understood when considering the different time scales involved: if the biexciton has a significant probability to decay through a loss channel before the desired photon is emitted, then the quantum efficiency with which the emission can be steered into the desired channel is reduced.

To quantify the effect on our scheme, we have implemented an additional loss term into the model for the radiative loss:

$$\mathcal{L}_{rad}(\rho_s) = -\frac{\gamma_{rad}}{2} \sum_{i=X_H,X_V} \left(\mathcal{L}_{|G\rangle\langle i|} + \mathcal{L}_{|i\rangle\langle B|}\right)(\rho_s) \qquad (1)$$

with

$$\mathcal{L}_{\sigma}(\rho_s) = \left(2\sigma\rho_s\sigma^\dagger - \sigma^\dagger\sigma\rho_s - \rho_s\sigma^\dagger\sigma\right) \qquad (2)$$

Supplementary Fig.6 shows the cavity photon population for the system parameters of Fig.2 of the main text for optimum pulse frequency and varying radiative decay. Very similar results are obtained for the case of Fig.4. For the realistic parameters used here, the process is efficient well beyond a radiative decay of 150ps (~30μeV). Biexciton lifetimes up to 1ns (~4μeV) have been

observed experimentally, e.g., [3, 4]. We would like to emphasize that the data in Supplementary Fig.6 show the total photon population, including any background emission from the single-photon transitions. If spectral filtering was used in photon detection, e.g., [5], the timescale limitation would be significantly alleviated, as the desired photon can be detected in a spectral region free of background photons. In summary, as expected radiative decay through other channels reduces the quantum efficiency and potential brightness of the source, however, the general emission scheme is not affected and also including radiative decay is efficient for realistic parameters at low temperatures.

Supplementary References:

[1] Bayer, M., Ortner, G., Stern, O., Kuther, A., Gorbunov, A. A., Forchel, A., Hawrylak, P., Fafard, S., Hinzer, K., Reinecke, T. L., Walck, S. N., Reithmaier, J. P., Klopf, F. & Schäfer, F. Fine structure of neutral and charged excitons in self-assembled In(Ga)As/(Al)GaAs quantum dots, Phys. Rev. B 65, 195315 (2002).

[2] Laucht, A., Hauke, N., Villas-Boas, J. M., Hofbauer, F., Kaniber, M., Böhm, G. & Finley, J. J. Dephasing of exciton polaritons in photoexcited InGaAs quantum dots in GaAs nanocavities, Phys. Rev. Lett. 103, 087405 (2009).

[3] Michler, P. Single Semiconductor Quantum Dots, Springer, Berlin (2009).

[4] Kim, H., Sridharan, D., Shen, T. C., Solomon, G. S. & Waks, E. Strong coupling between two quantum dots and a photonic crystal cavity using magnetic field tuning, Optics Express 19, 2589-2598 (2011).

[5] González-Tudela, A., del Valle, E. & Laussy, F. P. Optimization of photon correlations by frequency filtering, Phys. Rev. A 91, 043807 (2015).
